# HADDOCK3: A Modular and Versatile Platform for Integrative Modeling of Biomolecular Complexes

**DOI:** 10.1021/acs.jcim.5c00969

**Published:** 2025-06-17

**Authors:** Marco Giulini, Victor Reys, João M. C. Teixeira, Brian Jiménez-García, Rodrigo V. Honorato, Anna Kravchenko, Xiaotong Xu, Raphaëlle Versini, Anna Engel, Stefan Verhoeven, Alexandre M. J. J. Bonvin

**Affiliations:** † Bijvoet Centre for Biomolecular Research, Faculty of Science—Chemistry, 8125 Utrecht University, Padualaan 8, 3584 CH Utrecht, The Netherlands; ‡ Zymvol Biomodeling, Carrer de Pau Claris 94, 3B, 08010 Barcelona, Spain; § 443478 Netherlands eScience Center, Science Park 402, 1098 XH Amsterdam, The Netherlands

## Abstract

HADDOCK is a widely used resource for integrative modeling of a variety of biomolecular complexes that is able to incorporate experimental knowledge into physics-based calculations during complex prediction, refinement, scoring and analysis. Here we introduce HADDOCK3, the new modular version of the program, in which the original, parametrizable albeit rigid pipeline has been first broken down into a catalogue of independent modules and then enriched with powerful analysis tools and third-party integrations. Thanks to this increased flexibility, HADDOCK3 can now handle multiple integrative modeling scenarios, providing a valuable, physics-based tool to enrich and complement the predictions made by machine learning algorithms in the post-AlphaFold era. We present examples of successful applications of HADDOCK3 that were not feasible with the previous versions of HADDOCK, highlighting its expanded capabilities. The HADDOCK3 software source code is freely available from the GitHub repository (https://github.com/haddocking/haddock3) and comes with an online user guide (www.bonvinlab.org/haddock3-user-manual). All example data described in this manuscript are available at https://github.com/haddocking/haddock3-paper-data.

## Graphical abstract

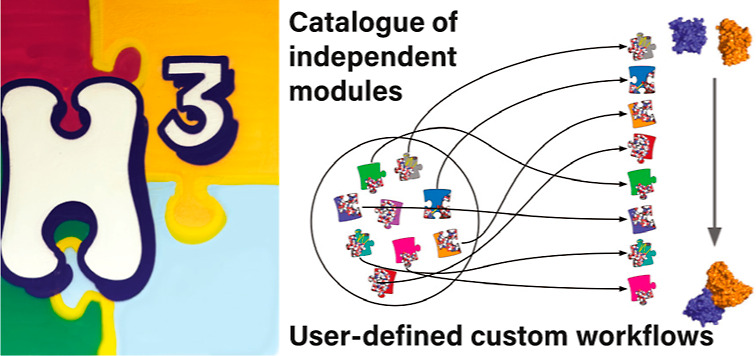

## Introduction

Modeling biomolecular interactions plays a central role in structural biology, as most biological processes take place through intricate networks of interactions occurring in the crowded cellular and extracellular environments.

In the last ten years, much progress has been made in the computational modeling of such interactions, in particular thanks to the development of deep learning-based methods able to accurately predict the three-dimensional structure of protein–protein complexes from sequences,^1–5^ recently expanded to the modeling of nucleic acids,^6^ as well as lipids, small molecules, and post-translational modifications.^7,8^

Classical approaches such as molecular docking are complementary to machine learning architectures,^9,10^ as they use physics-based methods to tackle challenging systems that cannot be accurately modeled using current machine learning methods. Although the typical success rate of these approaches is quite low when used in ab initio mode (i.e., with no a priori information), it can be substantially improved in data-driven mode, i.e., when some experimental knowledge is available about the interaction.^11–13^

Incorporating existing experimental information in the modeling process is at the core of the HADDOCK software,^11^ which uses the concept of ambiguous interaction restraints (AIRs) to bias physics-based docking toward models that maximize the consistency with the input data.

HADDOCK has been widely used in the last 20 years, with more than 60 000 registered users being able to perform almost 700 000 docking runs on the HADDOCK web server^9^ which has been in operation since 2008. The HADDOCK2.X series of software^9,14,15^ consists of a rigid, albeit highly parametrizable pipeline, in which rigid-body docking is followed by semiflexible interface refinement and a final energy minimization. Optionally, the final minimization can be replaced by a refinement in explicit solvent.

Here we introduce HADDOCK3, the next version of the HADDOCK software, which represents a complete rewriting and rethinking of the entire architecture. The original HADDOCK pipeline has been broken down into a catalogue of independent modules that can be, in large parts, freely combined into user- and system-specific custom workflows.

This modular and flexible architecture allows HADDOCK3 to handle a multitude of integrative modeling scenarios, providing a valuable, physics-based tool to enrich and complement the predictions made by machine learning algorithms in the post-AlphaFold era. In the following, we describe this new HADDOCK3 version and illustrate its capabilities with a few relevant examples, highlighting how workflows that were not feasible with the previous version can provide accurate structural models.

HADDOCK3 is an open-source software suite, freely available for download from our GitHub repository https://github.com/haddocking/haddock3 or directly from PyPI (https://pypi.org/project/haddock3). The user manual of HADDOCK3 can be accessed at www.bonvinlab.org/haddock3-user-manual.

## Software

### Bringing Modularity into HADDOCK

Modeling biomolecular interactions at the structural level typically involves several independent steps: docking the molecules together, refining the obtained models, and scoring them using various scoring functions. HADDOCK3 breaks down the fixed pipeline of the HADDOCK2.X series, thus allowing for a diversification of workflows where not all steps are required and where their order can be customized and optimized for each system under study. This is made possible by HADDOCK3’s modular structure, in which each module is an independent, self-sufficient step to be included in a larger modeling workflow, except for the required initial module that builds the molecular topology of the system. As a result, modules can be freely combined to address different scientific questions.

The modular design also simplifies the integration of new modules into the software, reducing compatibility issues. Name of parameters, their description, and allowed values for each module can be queried using the haddock3-cfg command line interface and found online at www.bonvinlab.org/haddock3.

Five categories of modules are currently available in HADDOCK3 (see Table 1):

- *topology modules*, responsible for the creation of topologies for the available modules;
- *sampling modules*, namely the traditional rigid-body docking methods (including HADDOCK2’s *it0* stage, now renamed rigidbody);
- *refinement modules*, in which the modeled systems are subject to various refinement algorithms, ranging from simple energy minimization (EM) to full explicit solvent molecular dynamics (MD) refinement (HADDOCK2’s *it1* now flexref and *itw*, now split into mdref and emref, are part of this category);
- *scoring modules*, in which models are ranked according to various scoring functions;
- *analysis modules*, encompassing a broad class of analysis methods, including model evaluation, clustering and analysis and visualization of interface properties.

**Table 1:** List of the Currently Available Modules in HADDOCK3, Each One with Its Own Category, Description, and Corresponding Analogue in the HADDOCK2.x Series, When Applicable

module name	category	function	HADDOCK2.X analogue
topoaa	topology	topology generation	begin
rigidbody	sampling	rigid-body docking of the molecules	*it0* stage
lightdock	sampling	swarm-based docking software^12^	
flexref	refinement	semiflexible interface refinement of the models following a simulated annealing MD protocol in torsion angle space	*it1*
emref	refinement	EM refinement	*itw* without solvent
mdref	refinement	MD refinement in explicit solvent	*itw*
openmm	refinement	openMM molecular dynamics^16^	
emscoring	scoring	energy minimization scoring of models	
mdscoring	scoring	molecular dynamics-scoring of models	
prodigyligand	scoring	binding affinity prediction of protein–ligand complexes using PRODIGY-lig^17^	
prodigyprotein	scoring	binding affinity prediction of protein–protein complexes using PRODIGY^18,19^	
sasascore	scoring	solvent accessible surface area scoring	
alascan	analysis	alanine (or other mutations) scanning of interface residues	
caprieval	analysis	energetics and other features extractions and CAPRI^20^ analysis of the models with respect to the best model or a provided reference structure	partial analysis present on the web server only
clustfcc	analysis	fraction of common contacts clustering	FCC clustering
clustrmsd	analysis	RMSD-based clustering	RMSD clustering
contactmap	analysis	contact map analysis	
filter	analysis	filter models based on score threshold	
ilrmsdmatrix	analysis	interface ligand RMSD matrix calculation	RMSD matrix calculation
rmsdmatrix	analysis	RMSD matrix	
seletop	analysis	selection of a number of models	sampling parameters
seletopclusts	analysis	selection of a number of models from a number of clusters	

### Workflow Definition

HADDOCK3 defines workflows using a configuration file compatible with the TOML format but with additional features. In the first part of this file, global parameters are specified, such as the input molecules, the running mode, and the output path. This is followed by the sequence of modules to be executed, each with several parameters that can be fine-tuned or left at their default settings.

Figure 1 shows the direct translation of the HADDOCK2 pipeline into a HADDOCK3 configuration file. Information to guide the docking is provided to the workflow through ambiguous interaction restraints^11^ (ambig_fname), ensuring that the resulting models align with the available experimental data. Additionally, unambiguous restraints (unambig_fname) can be added to fix the distance between specified pairs of atoms, which is particularly useful when modeling antibody–antigen complexes or dealing with MS cross-link data. The restraint files need to be specified for each module separately, which now gives more flexibility in the definition of which restraints are being used at each stage of the workflow.

**Figure 1:**
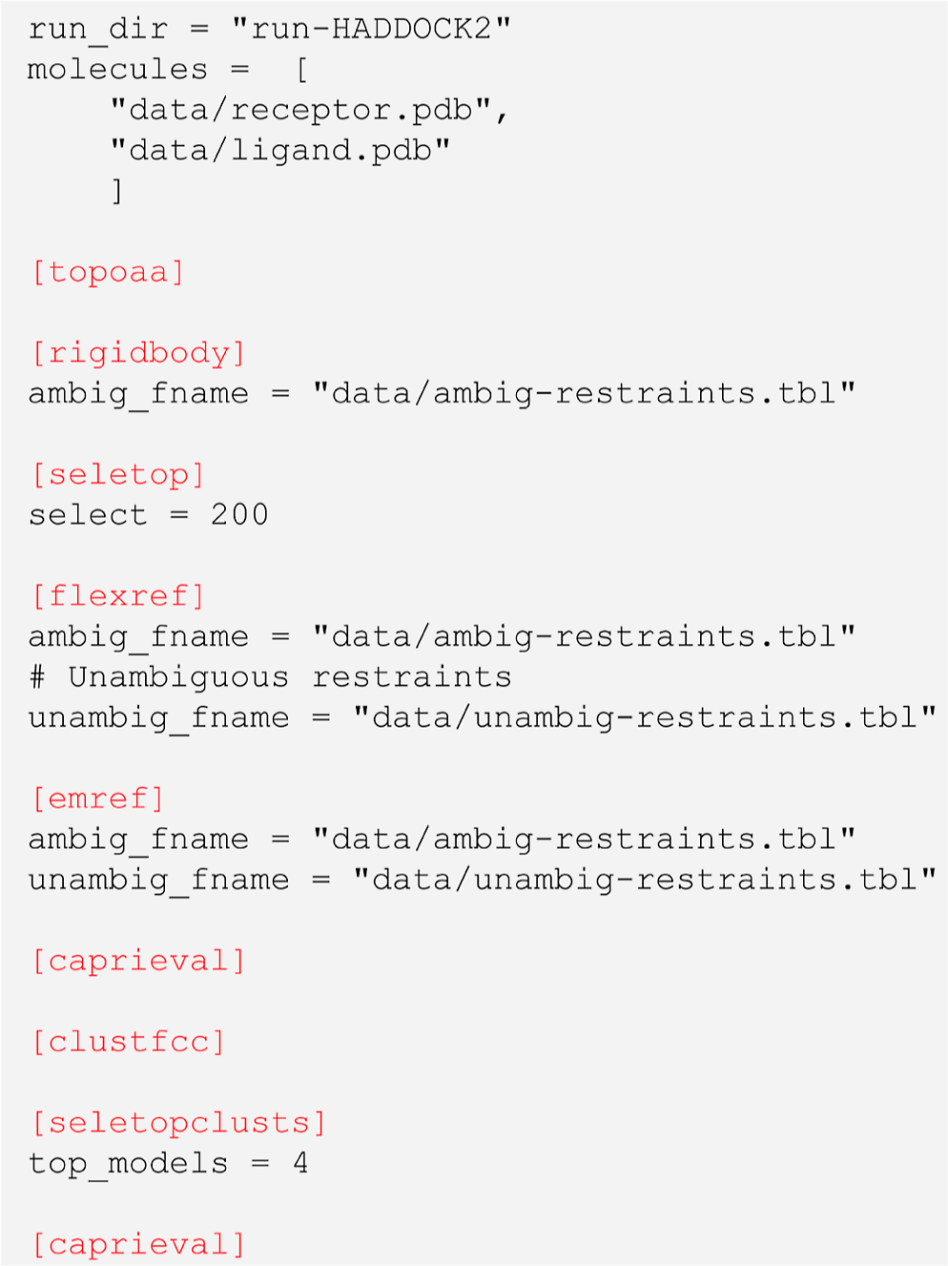
Literal translation of the HADDOCK2 pipeline into an HADDOCK3 workflow. After defining some preliminary global parameters such as the name of the directory where to perform the docking run (run_dir) and a list of paths to the input molecules (molecules), the list of modules defining the workflow is executed sequentially. First, the all-atom topology is built (topoaa). Then, the rigidbody module (former *it0*) docks the molecules according to the information contained in the ambiguous interaction restraints. A thousand models are generated at this stage by default, but only 200 of them (the ones with the lowest score) are taken to the next step of the workflow (seletop), namely the computationally more costly interface refinement (flexref). Models are then refined through energy minimization (emref) and their statistics extracted through the caprieval module, which performs a quality assessment of the models against the top ranked model. Models can be compared instead to a reference structure, if available, by defining the target name. They are then clustered based on the fraction of common contacts^21^ (clustfcc). seletopclusts subsequently selects the top 4 models of each cluster. Lastly, an additional caprieval analysis is performed again to obtain cluster-based statistics.

### Workflow Execution

HADDOCK3 is a command line interface (CLI) software that requires the user to use a terminal to launch a docking run. Once HADDOCK3 is installed, the path to the workflow configuration file must be passed as input to the haddock3 CLI to execute a run.

### Workflow Results

At execution time, HADDOCK3 will process a workflow in several steps. First, the content of the workflow configuration file is analyzed and checked for compliance with the existing modules and parameters. Input file paths are verified, module names and their related parameters are matched against available ones. Once this validation is passed, the run directory is created and input files are stored in the data directory. As a second step, the workflow modules are executed in a stepwise manner. Each module will create its own directory (numbered sequentially) where generated output files will be written.

Upon workflow completion, two postprocessing steps are performed by default: first, an analysis phase generates various plots that describe the produced models with their HADDOCK scores and components. Distributions of different metrics used in the Critical Assessment of PRotein Interactions (CAPRI)^20^ (ligand-rmsd, interface-ligand-rmsd, fraction of native contacts, and DockQ^22^) are reported, with comparisons made against the best scoring model or a provided reference structure. Then, a traceback step simplifies the analysis of the workflow’s progression, allowing users to trace how models evolved through the various modules and which input conformations or restraint files were used.

### Optimizing Execution Modes to Fit User Setups

Being able to run HADDOCK3 on various operating systems and hardware, increasing interoperability and scale, was a guideline of the development of this new version. HADDOCK3 is written in python3, leveraging an easy installation procedure on most operating systems through pip install commands. Detailed installation instructions are provided on the HADDOCK3 GitHub repository (https://github.com/haddocking/haddock3). A dedicated parameter (mode) enables the user to specify the best execution mode for the system on which the workflow will be run. Three execution modes are available:

- Local mode: HADDOCK3 runs on the current system, using a specified number of cores, which is limited by the available resources. This mode can also be used to, for example, run HADDOCK3 using a full node in a HPC environment.
- Batch mode: HADDOCK3 is initiated from a local server, using the system batch submission system (SLURM or torque) to submit short jobs to a defined queue.
- MPI mode: this mode allows modeling workflows to span multiple nodes on an HPC architecture, thus enabling distributed computing (provided MPI libraries are installed).

### HADDOCK3 Tools

HADDOCK3 comes with several command line interfaces (CLIs), each with its specific goal. The most important ones are described below, but the full list of command line interfaces can be found in the online user manual at www.bonvinlab.org/haddock3-user-manual/clis.html.

- The haddock3 command is the main CLI allowing to start a HADDOCK3 workflow.
- The haddock3-cfg CLI is made to retrieve and list the parameter names, their description, and default values for each available module.
- The haddock3-analyze CLI was developed to postprocess a docking run and generate tables and plots describing the complexes.
- The haddock3-restraints CLI is dedicated at creating and manipulating ambiguous restraints that are used in HADDOCK.
- The haddock3-score is a scoring CLI, that seamlessly performs topology generation, short energy minimization and scoring with the HADDOCK score of the input complex. It bypasses the need of generating a configuration file for the scoring of a single complex.

## Use Cases

In this section we describe four use cases that highlight HADDOCK3 workflows tailored to specific scientific problems that were not possible to address with the previous versions of the software.

### Targeting Multiple Interfaces

Information-driven modeling of biomolecular complexes requires some knowledge about the interface in order to be effective. In a typical two-body docking scenario, information may be only available for one of the molecules, while the binding site on the partner is ambiguous, meaning that the experimental (or bioinformatics) data suggest several possible binding interfaces.

In previous versions of HADDOCK, two main strategies have been adopted to tackle this quite common scenario. In the first one, two or more separate docking runs are performed, each one targeting a specific interface. A posteriori comparison based on visual inspection and other analysis tools were then used to select the most likely correct binding interface. In the second one, different solvent-accessible patches are defined together as passive (passive in HADDOCK context means that a given residue can be at the interface), hoping that the random removal of restraints and their ambiguity would allow obtaining near-native docking poses. Both strategies are suboptimal, as they rely either on parallel computations and cumbersome additional analysis or on a possibly physically implausible combination of sets of restraints. In HADDOCK3 this problem is addressed by adding the possibility to provide several sets of restraints within a single workflow. The initial rigid body docking models are generated using different sets of restraints. Information about which set was used is propagated for each model to all subsequent stages of the workflow. The scoring function will compare them within the HADDOCK machinery, therefore eliminating the need for multiple parallel runs and a posteriori analysis.

Here we present an example of such an application for the modeling of an antibody–antigen complex, namely the complex (PDB ID 4G6M)^24^ between gevokizumab and interleukin-1β (IL-1β). Using ARCTIC-3D,^23^ we automatically extracted from the PDB Knowledge base^25^ the five interfaces (epitopes) formed by IL-1β with different antibodies, including the one specific to gevokizumab (see Figure 2a). Using the solvent-exposed residues on the complementarity-determining region (CDR) of the unbound antibody structure (PDB ID 4G6K),^24^ we generated five sets of ambiguous interaction restraints, each one effectively targeting a different epitope.

**Figure 2:**
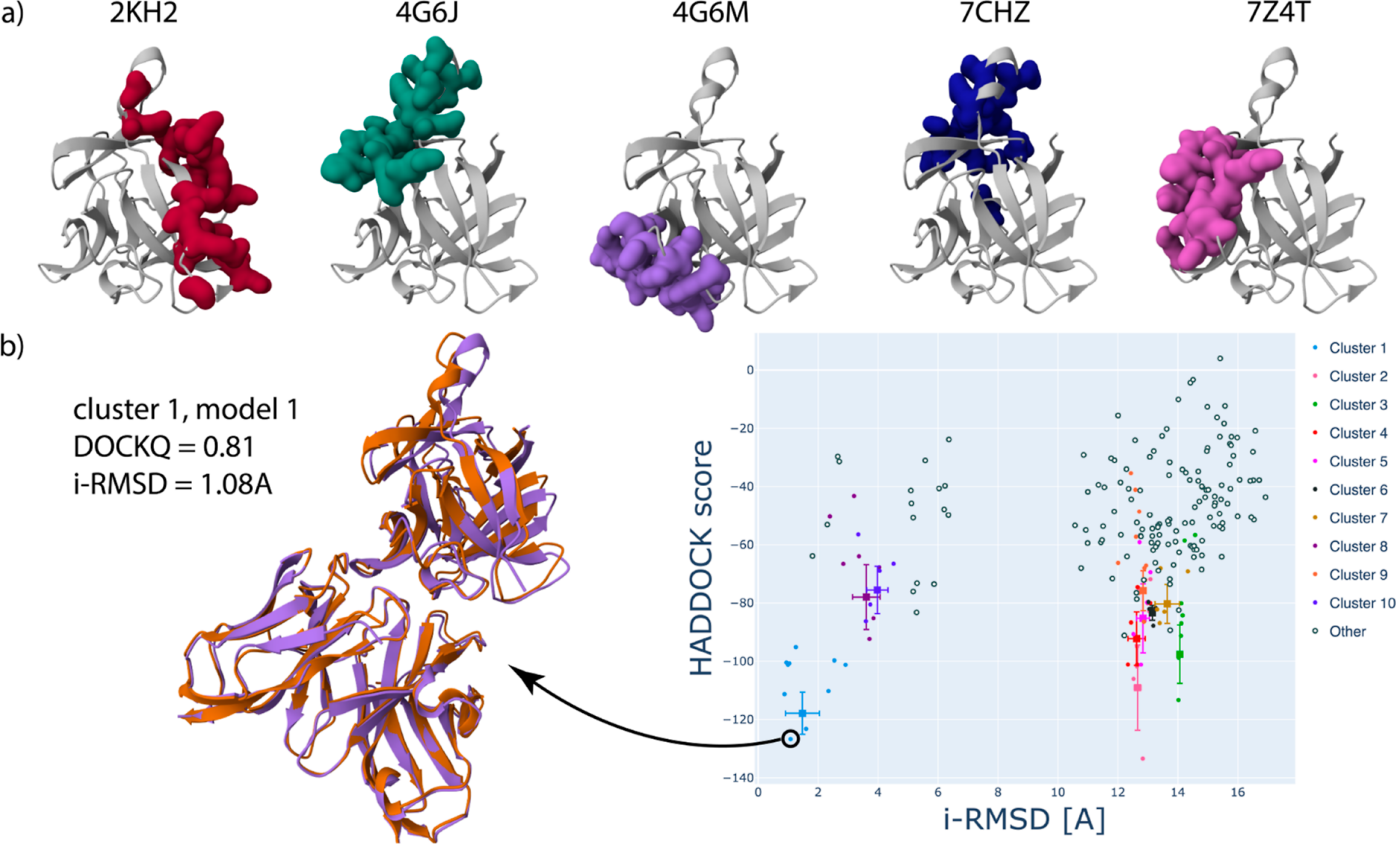
Illustration of multiple restraints targeting different binding sites. (a) ARCTIC-3D^23^ analysis reveals five different epitopes in interleukin-1β. The PDB ID corresponding to each epitope is reported and the amino acids belonging to it are shown in green on the unbound structure of interleukin-1β (coming from PDB ID 5R8Q, chain B). (b) Results of a HADDOCK3 run targeting multiple interfaces within the same workflow. The best model of the top-ranked cluster is shown overlaid on the reference structure (PDB ID 4G6M), highlighting the excellent agreement with the model (DOCKQ = 0.81). On the right, a HADDOCK3 scatter plot between the HADDOCK score and the interface RMSD illustrates how the best cluster can be clearly identified.

A standard HADDOCK workflow is then executed. When considering the cluster-based results, we see how the best cluster is targeting the correct interface and is composed of models of medium-to-high quality following CAPRI criteria, with DockQ values ranging from 0.48 to 0.88 (Figure 2b). For a comparison, we run a HADDOCK workflow targeting the five different epitopes at the same time (defined together as passive residues, see above). Here HADDOCK cannot generate any acceptable model, with the top-ranked model and overall best model (ranked 136) displaying values of DockQ of 0.034 and 0.072, respectively. In this case, the information encoded in the ambiguous restraints is too sparse and generic, hindering the identification of the correct binding mode of the antibody.

This example demonstrates that HADDOCK3’s ability to handle multiple restraint sets in a single run can effectively identify the correct binding interface with high-quality models.

### Protein–Glycan Docking: Introducing a Clustering Step after Rigid-Body Docking

Glycans are very flexible biomolecules formed by two or more monosaccharides linked by glycosidic bonds. The chemical composition and branching pattern of such linkages create an incredibly diverse set of possible oligosaccharides.^26^

The flexibility provided by HADDOCK3 is particularly suited to model noncovalent protein–glycan interactions, especially when information is available on the protein binding site. In a recent study^27^ we demonstrated that tweaking the original HADDOCK recipe provides a substantial increase in the docking performances, reaching a success rate of 60% when considering the top 10 models. Two major ingredients lie at the core of this improvement, namely the increased weight assigned to the hydrophobicity (van der Waals) component of the HADDOCK energy function, and the insertion of a fine-tuned RMSD clustering step between the sampling (rigidbody module) step and the flexible interface refinement (flexref module). This was not possible in the previous version of HADDOCK due to its predefined, fixed pipeline.

We illustrate this slightly more complex workflow in Figure 3, which we use to model the complex between rainbow trout lysozyme and a linear oligosaccharide (PDB ID 1LMQ).^28^ The oligosaccharide consists of three 2-acetamido-2-deoxy-β-d-glucopyranose and one 2-acetamido-2-deoxy-α-d-glucopyranose, with β 1–4 linkages connecting the four monosaccharide units. We use PDB ID 1LMN as the unbound^29^ conformation of the protein, while the conformation of the glycan was generated with the GLYCAM Web server (https://glycam.org).^30^ The protein closely resembles its bound form, with a backbone RMSD of 0.46 Å, while the modeled oligosaccharide does not fully align with the solved bound structure, especially in the ring conformation of 2-acetamido-2-deoxy-α-d-glucopyranose with a heavy atom RMSD between the two glycan conformations of 1.35 Å. Ambiguous interaction restraints are defined between the interface amino acids of the protein (defined as active) and the entire glycan (defined as passive).

**Figure 3:**
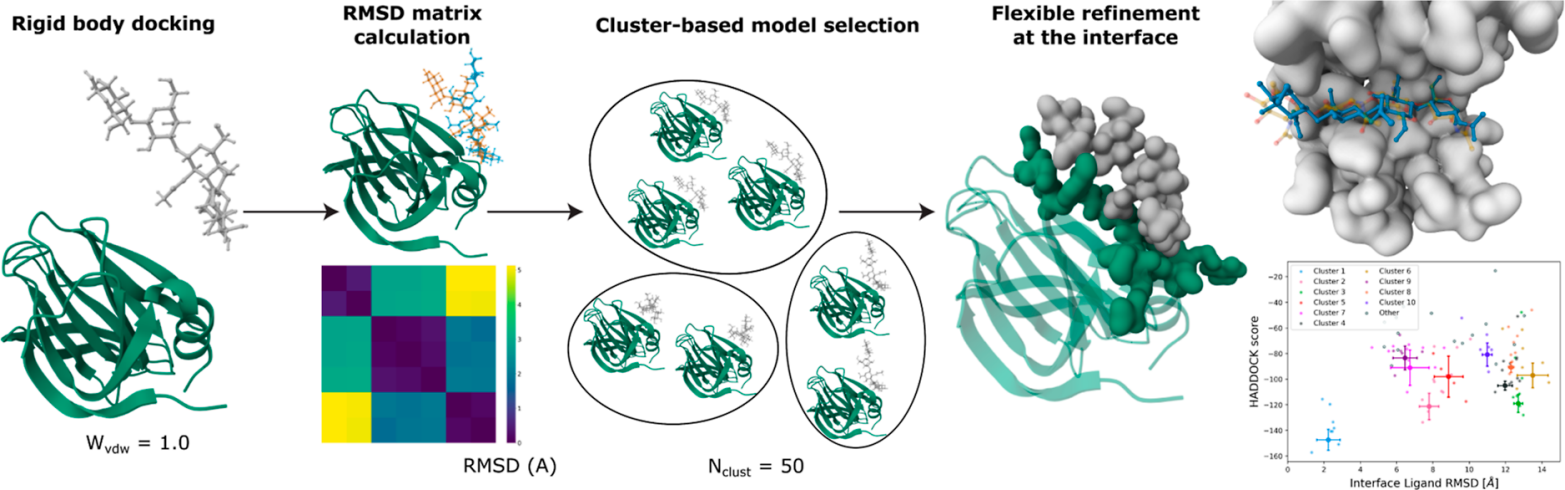
Workflow scheme for the protein–glycan modeling pipeline with a clustering step after rigid-body docking. The models that are chosen in this cluster-based selection are then subjected to a flexible refinement of the interface, and again clustered and analyzed. Figure adapted with permission from ref 27.

When running the standard HADDOCK workflow, the best-scored docking models display values of DockQ around 0.47, with Interface-ligand RMSD (IL-RMSD) between 6 and 7 Å. The new docking workflow where RMSD clustering is performed between rigid-body docking and flexible refinement stages gives significantly better results, with the best cluster showing a mean DockQ of 0.83 and a mean IL-RMSD equal to 2.24 Å in its top four models. These markedly improved results are due to the clustering step, which performs a selection based on conformational diversity rather than purely selecting models according to the HADDOCK scoring function. Indeed, at the rigid-body docking step, the accurate models are not ranked among the top 200 in this case, with the best model (DockQ = 0.70) being ranked in position 947 out of 1000 sampled structures.

This example highlights that adding intermediate clustering steps and performing a cluster-based selection of docking models can significantly improve the docking results at the end of the workflow, especially when one or more input molecules are not modeled with high accuracy.

### Consensus Scoring

Scoring models of biomolecular complexes is one of the core tasks of an integrative modeling platform, as it allows, if successful, to discriminate between near-native and non-native complexes. The scoring experiment has been included in the CAPRI challenge since 2005.^31^ In this context, research groups are asked to rank an ensemble of typically a few thousand models for each complex, to select the best 5 or 10 models. The scoring is considered successful if at least one good model, according to CAPRI criteria, is present in this reduced set.

A plethora of scoring functions have been developed over the recent years,^32–36^ each one with its own strengths and weaknesses.

Applying multiple scoring functions to the same set of complexes can be beneficial to improve ranking accuracy.^34,37,38^ The HADDOCK3 suite is perfect for this task, as its modularity allows users to fine-tune scoring workflows by sequentially applying various types of scoring functions and inspecting the final consensus results. Five scoring modules are already available in the software, with the possibility to easily add more of them by exploiting HADDOCK3’s modularity. Additional clustering and analysis steps may be added to such workflows to group similar models together and to visualize their features.

We tested this approach in CAPRI round 57, where the HADDOCK3 energy minimization and scoring were successfully combined with the VoroIF-jury method.^34^ Here we focus on Target 268, an antibody–peptide complex, for which HADDOCK3 not only submitted the best model across the whole CAPRI scoring set (DockQ = 0.72, high-quality model according to CAPRI criteria) but also ranked it in the first position of its scoring ensemble. Other two scorer groups (Olechnovič^34^ and LZERD^39^) were able to submit a high-quality model in the reduced scoring set of five complexes, although with a slightly lower quality (DockQ = 0.69).

Figure 4a shows the consensus scoring procedure performed throughout CAPRI round 57. The models to be scored are provided as a single PDB file containing an ensemble of models (with the MODEL/ENDMDL construction). The workflow begins with building the topology of the input models (topoaa), followed by a short energy minimization step after which the HADDOCK scoring function is applied (emscoring). Next, FCC clustering^21^ (clustfcc module) is performed and the top four models of the best ten or 15 clusters are selected for the final evaluation (seletopclusts). Finally, the VoroIF-jury method (embedded in the voroscoring module) is applied to provide an alternative scoring function. In Figure 4b we show the relationship between the two scoring functions applied to CAPRI target 268: according to VoroIF-jury, both cluster 1 and cluster 4 are reasonable choices, but the HADDOCK scoring function helps remove this ambiguity by assigning a significantly better score to cluster 1.

**Figure 4:**
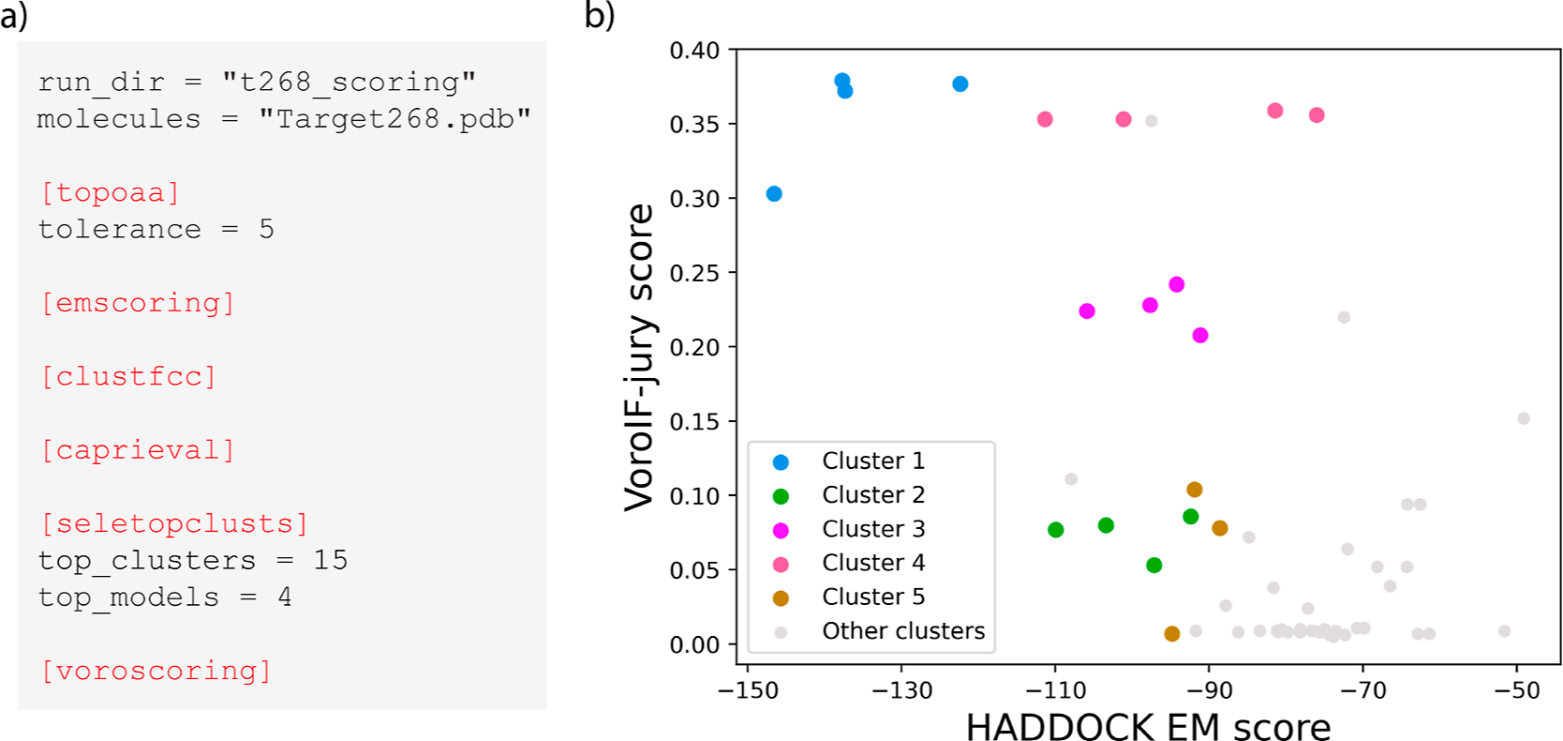
Illustration of HADDOCK3 scoring. (a) The HADDOCK3 scoring workflow executed during CAPRI round 57. (b) Scatter plot between HADDOCK EM score (*x* axis) and VoroIF-jury score (*y* axis) for CAPRI target 268. The four models belonging to the top five FCC clusters are highlighted in color, while models that are part of other clusters (from cluster 6 to cluster 15) are shown in gray. Models from cluster 1 stand out unambiguously in the top left corner (best—most negative—HADDOCK score and highest VoroIF-jury score). The best model in terms of energetics has a slightly lower VoroIF-jury score than the other cluster members.

This is only one example of the possibility to develop advanced consensus scoring workflows within the modular HADDOCK3 framework, for example combining general methods, such as the energy-based HADDOCK score, with functions more tailored to specific biomolecular complexes.

### Analysis of Complexes and Detection of Hot Spots

The analysis of a complex, either experimentally determined or predicted, can bring useful information about the residues involved in the interaction, to detect hot spots, and predict the potential impact of point mutations. In HADDOCK3, several analysis modules are available to inspect and describe biomolecular interactions. Among them, the contact-map (contactmap) module is dedicated to analyzing contacts present in complexes and rendering them as both heat maps and chord charts (Figure 5c,d). Interactive plots are generated using the *plotly* library,^40^ enabling a smooth navigation and understanding of the contacts in a given complex. In the case of a HADDOCK workflow with a clustering step, one plot per cluster will be generated, allowing for a fast identification of the different contact patches belonging to the various clusters.

**Figure 5:**
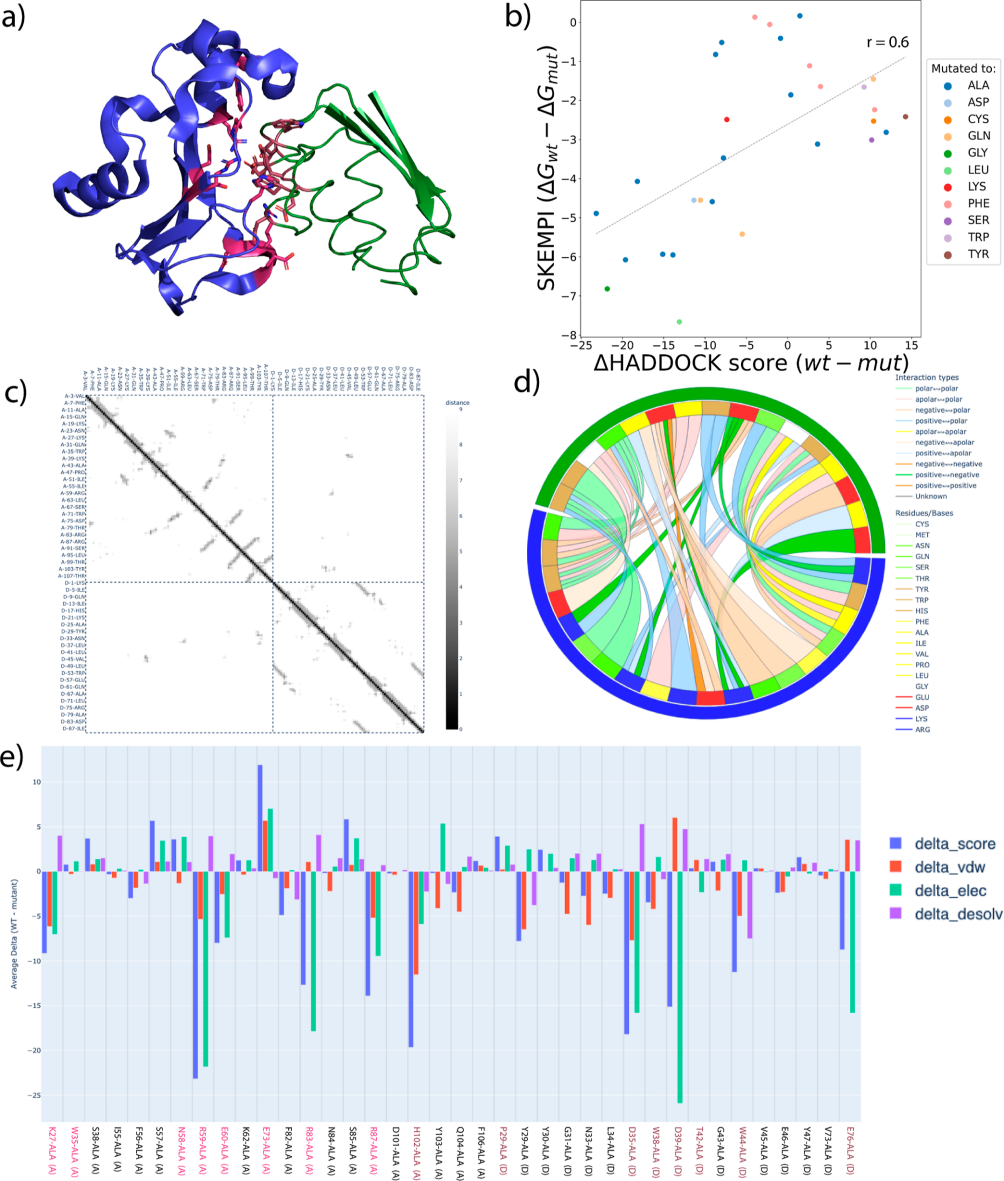
Interaction analysis with HADDOCK3. (a) PyMOL^41^ representation of the barnase–barstar complex (PDB ID 1BRS), where residues mutated present in the SKEMPI database are colored in red. (b) Scatter plot of the difference in HADDOCK score (score wild-type—score mutant) obtained using the alascan module and difference in Gibbs free energy from SKEMPI (Δ*G*_wt_ – Δ*G*_mut_) on the barnase–barstar complex for single point mutations only. A Pearson correlation coefficient of 0.6 is obtained for this complex. (c) Heat map, generated by the contactmap module, of the contacts observed on the PDB structure 1BRS. (d) Chord chart, generated by the contactmap module of the interchains contacts observed on the PDB structure 1BRS. (e) alascan plot displaying the influence of interface residues mutated to alanine. Colored labels are present in the SKEMPI data set.

Another useful feature is provided by the alanine scanning (alascan) module, which detects residues at the interface of a complex and iteratively mutates them to alanine (default, but other mutations can be specified). The alascan module then performs a short energy minimization and computes the HADDOCK score of the mutated complexes. For each amino acid at the interface (defined using a 5 Å cutoff between any heavy atom of residues belonging to different chains), the module reports the difference in HADDOCK score between the wild-type sequence and the mutant, as well as the contribution of each component of the score (van der Waals, electrostatics, and desolvation energies). Once again, an interactive plot can be generated to obtain a graphical representation of the various energetical contributions of the interface residues (Figure 5b) and possibly identify hot-spots. Although this module holds the name alascan, the conversion to any other amino acid is possible, thus providing a physics-based method to screen mutations in protein design projects.

Here we show a combination of these analysis tools applied to the barnase–barstar complex (PDB ID 1BRS)^42^ using the workflow shown in Figure 6.

**Figure 6:**
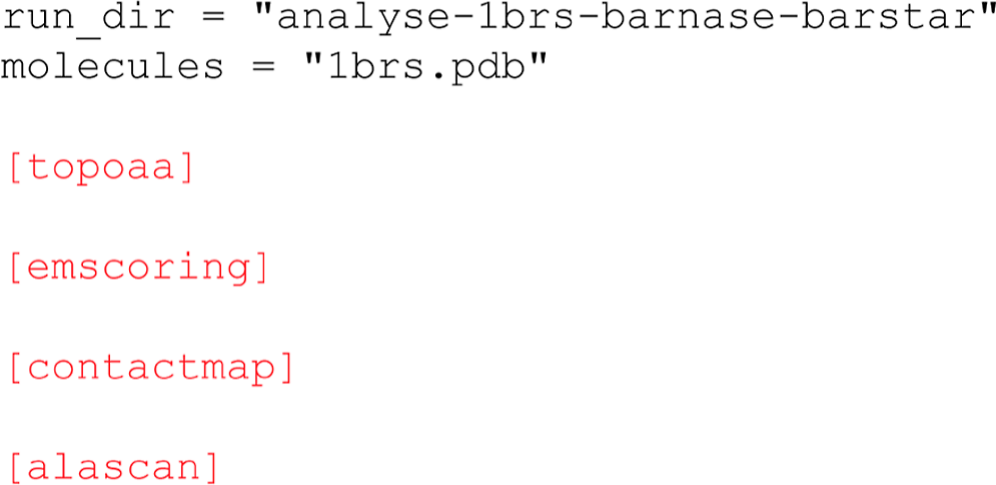
Example workflow for the analysis of a complex. Here the input complex (1brs.pdb) is provided as a single molecule and subjected to a short energy minimization. The contactmap and alascan modules perform contact-map and alanine scanning analysis of the model, respectively (see main text).

After a short minimization of the crystal structure, the intra and inter chains contacts are visualized as a heat map (Figure 5c) by running the contactmap module, which also generates a chord chart coloring the contacts by type (Figure 5d). Finally, the alascan module performs a systematic mutagenesis to alanine of all interface residues. The resulting changes in HADDOCK score and its components are plotted (Figure 5e). For this system, experimental binding affinity data are available from the SKEMPI database.^43^ We extracted all single point values (ddG) and compared those to the changes in HADDOCK score from the alascan module (Figure 5b). In this case, and without any optimization, we observe a Pearson correlation of 0.6 between the experimental ddG and difference in HADDOCK score, showing that the HADDOCK score, together with the alascan module, can be used to obtain information about the influence of a given residue mutation. Note however that these observations are rather specific to the barnase–barstar complex, which has quite a hydrophilic/polar interface. From experience, correlations are expected to be worse for more hydrophobic interfaces. We refer to our previous work on predicting changes in binding affinities upon mutations for a more systematic analysis.^44,45^

## Conclusions

In this work we have introduced HADDOCK3, the new version of the integrative modeling software HADDOCK, which has been redesigned and modularized to provide higher flexibility and versatility in creating custom, system-specific modeling workflows. HADDOCK3 can seamlessly complement and assist machine learning-based structure prediction methods by incorporating available experimental data and physicochemical features of the studied systems. A few possible applications of the method have been showcased, such as the flexible modeling of antibody–antigen complexes targeting multiple potential binding sites, clustering-enhanced protein–glycan interaction prediction, the consensus scoring of user-provided structural ensembles, and the analysis of intermolecular interactions.

HADDOCK3 comes with several external online resources facilitating the training of new users with tutorials (www.bonvinlab.org/education/HADDOCK3), tracking of potential code-related issues (https://github.com/haddocking/haddock3/issues), providing day-to-day support to users (ask.bioexcel.eu) and accessing both the user manual (www.bonvinlab.org/haddock3-user-manual) and code documentation (www.bonvinlab.org/haddock3). In addition, on a yearly basis, a survey is conducted, where users are asked to give feedback and can request new features. This provides a basis for defining guidelines for the implementation of future features.

In conclusion, HADDOCK3, now easily available as a python package through PyPI, is a versatile and comprehensive software suite offering a wide range of options to address complex challenges in structural biology. We foresee that its new modular architecture will attract external software developers and users to contribute new modules and share workflows.

## Data Availability

Data, scripts, and HADDOCK3 configuration files used and described in this paper are available online on a dedicated GitHub repository hosted at https://github.com/haddocking/haddock3-paper-data.
